# The expression of interleukin-32 is activated by human cytomegalovirus infection and down regulated by hcmv-miR-UL112-1

**DOI:** 10.1186/1743-422X-10-51

**Published:** 2013-02-12

**Authors:** Yujing Huang, Ying Qi, Yanping Ma, Rong He, Yaohua Ji, Zhengrong Sun, Qiang Ruan

**Affiliations:** 1 Virus Laboratory, the Affiliated Shengjing Hospital, China Medical University, 110004, Shenyang, Liaoning, People’s Republic of China

**Keywords:** IL-32, HCMV infection, hcmv-miR-UL112-1

## Abstract

**Background:**

Interleukin-32 (IL-32) is an important factor in innate and adaptive immune responses, which activates the p38MAPK, NF-kappa B and AP-1 signaling pathways. Recent reports have highlighted that IL-32 is regulated during viral infection in humans.

**Methods:**

Enzyme-linked immunosorbent assays (ELISA) were carried out to detect IL-32 levels in serum samples. Detailed kinetics of the transcription of IL-32 mRNA and expression of IL-32 protein during human cytomegalovirus (HCMV) infection were determined by semi-quantitative RT-PCR and western blot, respectively. The expression levels of hcmv-miR-UL112-1 were detected using TaqMan® miRNA assays during a time course of 96 hours. The effects of hcmv-miR-UL112-1 on IL-32 expression were demonstrated by luciferase assay and western blot, respectively.

**Results:**

Serum levels of IL-32 in HCMV-IgM positive patients (indicating an active HCMV infection) were significantly higher than those in HCMV-IgM negative controls. HCMV infection activated cellular IL-32 transcription mainly in the immediately early (IE) phase and elevated IL-32 protein levels between 6 and 72 hours post infection (hpi) in the human embryonic lung fibroblast cell line, MRC-5. The expression of hcmv-miR-UL112-1 was detected at 24 hpi and increased gradually as the HCMV-infection process was prolonged. In addition, it was demonstrated that hcmv-miR-UL112-1 targets a sequence in the IL-32 3^′^-UTR. The protein level of IL-32 in HEK293 cells could be functionally down-regulated by transfected hcmv-miR-UL112-1.

**Conclusions:**

IL-32 expression was induced by active HCMV infection and could be functionally down-regulated by ectopically expressed hcmv-miR-UL112-1. Our data may indicate a new strategy of immune evasion by HCMV through post-transcriptional regulation.

## Background

Interleukin-32 (IL-32) is a newly-discovered pro-inflammatory cytokine, which plays a role in innate and adaptive immune responses [1,2]. It lacks sequence homology to any presently known cytokine families. IL-32 is associated with the induction of inflammatory responses by activating the p38MAPK, NF-kappa B and AP-1 signaling pathways. It has been implicated in inflammatory disorders, mycobacterium tuberculosis infections and inflammatory bowel disease, as well as in some autoimmune diseases, such as rheumatoid arthritis, ulcerative colitis and Crohn’s disease [3-10]. Moreover, it has been reported that IL-32 has pro-inflammatory effects on myeloid cells and promotes the differentiation of osteoclast precursors into multinucleated cells expressing specific osteoclast markers [11,12]. In recent studies, IL-32 has also been found to be regulated during viral infections. Elevated levels of IL-32 were found in sera from patients infected with influenza A virus [13-15], hepatitis B virus (HBV) [16], hepatitis C virus (HCV) [17], human papillomavirus (HPV) [18] and human immunodeficiency virus (HIV) [19-21], suggesting that IL-32 might play an important role in host defense against viral infections

Human cytomegalovirus (HCMV) is an ubiquitous β-herpesvirus that infects a broad range of cell types in human hosts, contributing to its complex and varied pathogenesis. HCMV Infection leads to life-long persistence in 50%–90% of the population, which is generally subclinical in healthy individuals [22]. However, it can lead to serious complications in immunocompromised patients, such as transplant recipients or AIDS patients [23,24].

MicroRNAs (miRNAs) are an abundant class of small non-coding RNA molecules that target mRNAs generally within their 3^′^ untranslated regions (3^′^UTRs). MiRNAs suppress gene expression mainly through inhibition of translation or, rarely, through degradation of mRNA [25,26]. Clinical isolates of HCMV encode at least 17 miRNAs [27,28]. However, only a few functional targets have been validated experimentally for some HCMV-encoded miRNAs [29-34]. It has been demonstrated that hcmv-miR-UL112-1 targets and reduces the expression of HCMV UL123 (IE1 or IE72), UL114 and the major histocompatibility complex class 1-related chain B (MICB). Moreover, BclAF1 protein, a human cytomegalovirus restriction factor, was reported to be a new target of hcmv-miR-UL112-1 [35]. In addition, multiple cellular targets of hcmv-miR-US25-1, which are associated with cell cycle control, were identified by RNA induced silencing complex immunoprecipitation (RISC-IP), including cyclin E2, BRCC3, EID1, MAPRE2 and CD147. IL-32, which is not present in the confirmed targets, was screened out as a candidate target of hcmv-miR-UL112-1 in our previous study [36]. However, no further experiments to validate these findings have been performed.

In the present study, the expression levels of IL-32 were compared among serum samples from patients with active HCMV infection and samples from HCMV-IgM negative individuals. The expression levels of IL-32 and hcmv-miR-UL112-1 in HCMV infected MRC-5 cells were detected at different stages of infection and time points. In addition, functional down-regulation of IL-32 by hcmv-miR-UL112-1 was detected in transfected human embryonic kidney (HEK293) cells, and the effect of hcmv-miR-UL112-1 on IL-32 during HCMV infection was primarily discussed.

## Results

### Relatively high IL-32 levels among individuals with active HCMV infection

**Figure 1:**
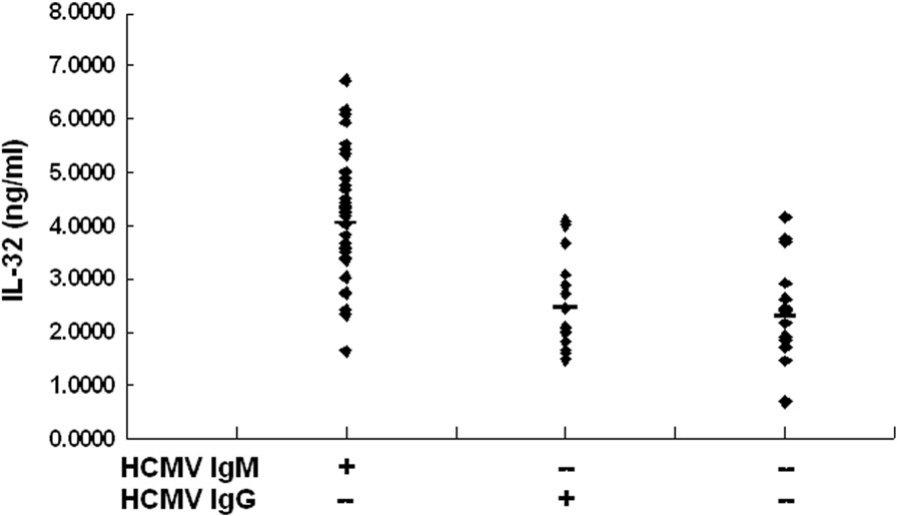
**An illustration showing IL-32 levels in serum samples detected by ELISA.** Serum samples were from actively HCMV-infected patients (n=40), HCMV previously infected individuals (n=17) and healthy individuals (n=15), respectively.

IL-32 protein levels were measured in the sera of 40 patients with active HCMV infections (HCMV IgM positive) and 32 HCMV IgM negative control individuals by enzyme-linked immunosorbent assays (ELISA). According to the results of HCMV IgG detection, HCMV IgM negative controls were divided into two groups: Previously HCMV infected group (IgG positive, n=17) and healthy group (IgG negative, n=15). Mean IL-32 levels in the sera of patients with active HCMV infection were 4.1778±1.1663 ng/ml (shown in Figure 1). Mean IL-32 levels in sera of the previously HCMV infected group and healthy group were 2.4653±0.8287 ng/ml and 2.4480±0.9162 ng/ml, respectively. The data were analyzed by *F* test*.* IL-32 levels in the sera of patients with active HCMV infection were significantly higher than those in the HCMV IgM negative control group (*F*=23.957, *P*<0.001). No significant difference was observed between the previously HCMV infected group and the healthy group (*P*=0.963).

### Kinetics of the expression of IL-32 mRNA, IL-32 protein and hcmv-miR-UL112-1 in HCMV infected MRC-5 cells

**Figure 2:**
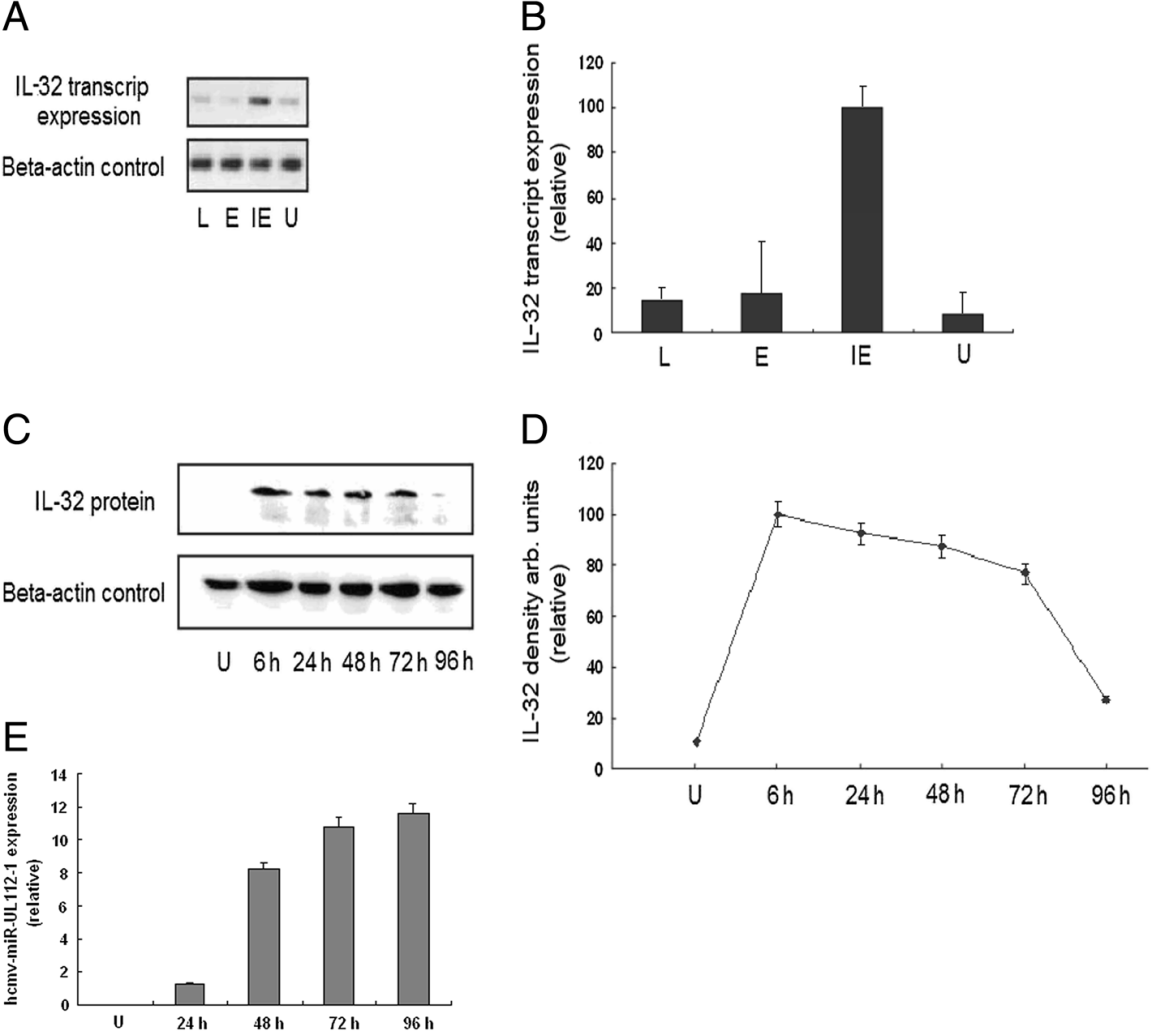
**Expression of IL-32 induced by HCMV infection in MRC-5 cells.** (**A**) IL-32 mRNA levels in HCMV infected cells were compared to that in uninfected MRC-5 cells. β-actin was used as an internal control. The universal primers for all IL-32 transcripts were used. U is for uninfected MRC-5; IE is for immediate early stage; E is for early stage; L is for late stage. (**B**) IL-32 mRNA levels were represented relative to that in IE stage. (**C**) IL-32 protein levels in HCMV infected MRC-5 cells were detected at different time points. IL-32 densitometer values were normalized by β-actin values. (**D**) The IL-32 protein levels were represented relative to that in infected cells collected at 6 hpi. (**E**) The kinetics of hcmv-miR-UL112-1 expression were measured using TaqMan® miRNA assays. The expression of hcmv-miR-UL112-1 could be detected at 24 hpi and increased gradually as the HCMV-infection process was prolonged.

Cells were harvested at different infection stages and time points after HCMV infection as described in the Materials and Methods section. We used specific drugs to determine the expression profile of IL-32 at different stages of viral replication. CHX was used to determine the immediate early (IE) stage and PAA was used to determine early (E) stage. As shown in Figure 2A and B, semi-quantitative RT-PCR analysis revealed that IL-32 mRNA expression was significantly activated at IE stage but not at E or late (L) stages post-infection. IL-32 protein expression levels at different time points following HCMV infection were measured by western blot analysis. As shown in Figure 2C and D, IL-32 protein expression reached a peak value at 6 hours post infection (hpi). The relative IL-32 concentration in HCMV infected cells collected at 6 hpi was 10 fold higher than in uninfected cells. All measured values were balanced by using β-actin as an internal control. No accumulation of either IL-32 mRNA or protein was found following the prolongation of HCMV-infection process. IL-32 mRNA levels in HCMV infected cells decreased in E and L stages to a similar level as in uninfected cells. IL-32 protein levels decreased with subsequent hours post infection.

In addition, the kinetics of hcmv-miR-UL112-1 expression were measured using TaqMan® miRNA assays. Measured values were normalized by using U6 as an internal control. The expression of hcmv-miR-UL112-1 could be detected at 24 hpi. As shown in Figure 2E, a steep increase was observed at 48 hpi. The relative quantity of hcmv-miR-UL112-1 at 48 hpi was 6.5 fold higher than at 24 hpi. The expression of hcmv-miR-UL112-1 increased gradually as the HCMV-infection process was prolonged. The relative quantity of hcmv-miR-UL112-1 in HCMV infected cells at 96 hpi was 10 fold higher than at 24 hpi.

### Functional down-regulation of endogenous IL-32 expression by ectopically expressed hcmv-miR-UL112-1

**Figure 3:**
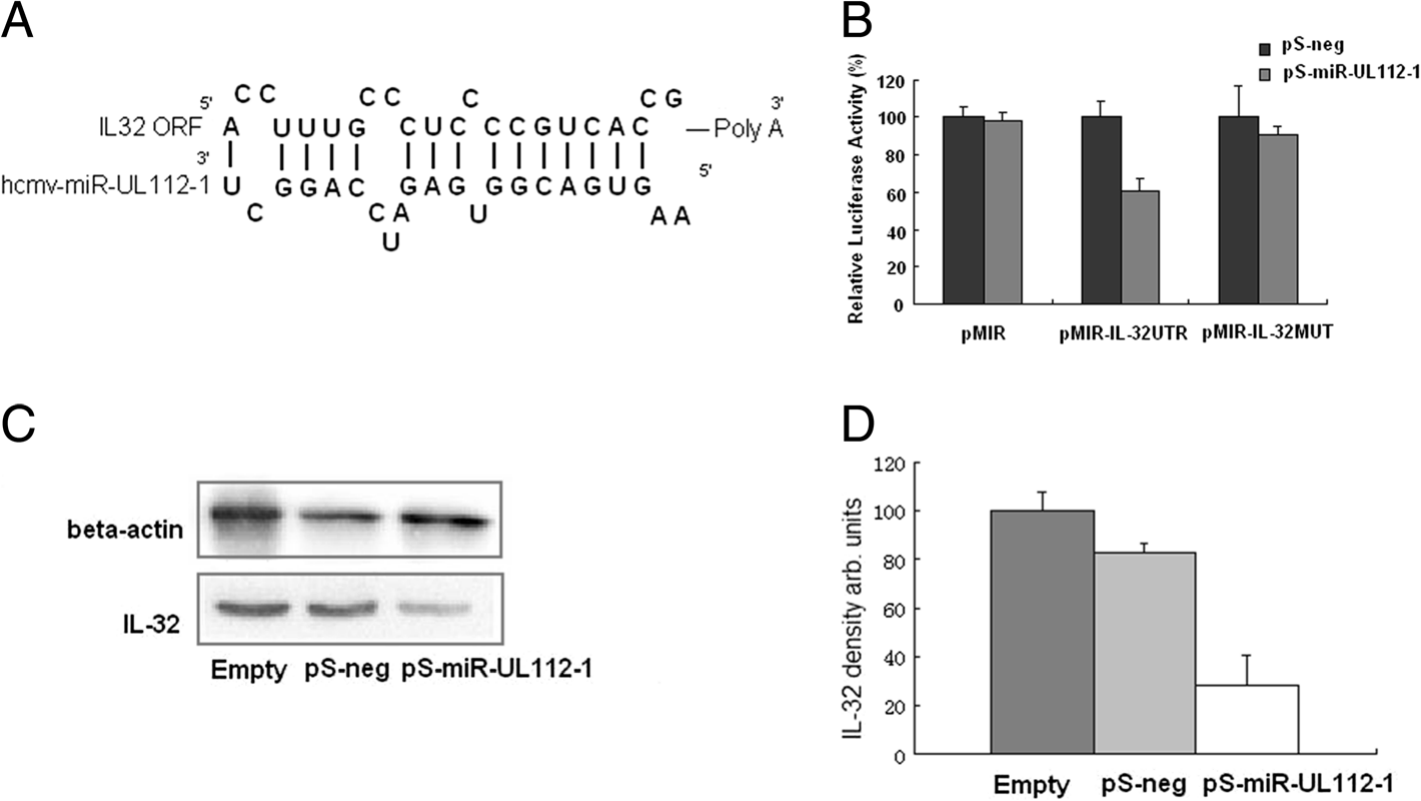
**Down-regulation of IL-32 expression by hcmv-miR-UL112-1.** (**A**) The diagram shows the predicted sequences of hcmv-miR-UL112-1 binding to IL-32 mRNA. (**B**) As a candidate target, IL-32 was validated for its ability to inhibit expression of a luciferase reporter construct in the presence of hcmv-miR-UL112-1 (pS-miR-UL112-1). Results are shown as percentage expression of negative control sample (pS-neg) following correction for transfection levels according to the control of renilla luciferase expression. Values are shown as means ± standard deviations for triplicate samples. (**C**) HEK 293 cells were transfected with pS-miR-UL112-1 or control vector respectively. Cells were collected 48 hpi and were subjected to western blot analysis using the indicated antibodies. IL-32 densitometer values were normalized to that of the β-actin values. (**D**) The amounts of proteins presented in panel C were quantified by densitometry. Results are shown as percentage expression of the negative control sample (Empty).

In our previous study, a sequence in the 3^′^UTR of IL-32 mRNA was identified by hybrid-PCR to be a candidate target site of hcmv-miR-UL112-1. The predicted binding site for hcmv-miR-UL112-1 was 195 nt upstream of the polyA structure of IL-32 mRNA. A schematic representation of hcmv-miR-UL112-1 binding to the IL-32 3^′^UTR sequence is shown in Figure 3A.

To further determine whether the IL-32 3^′^UTR sequence represents a functional target site for hcmv-miR-UL112-1, the IL-32 3^′^UTR sequence was validated by luciferase reporter assays. As shown in Figure 3B, transfected pMIR-IL-32UTR led to a significant inhibition of luciferase activity by 39.27% in the presence of pS-miR-UL112-1. pS-miR-UL112-1 had no significant inhibitory effects on the luciferase activities of pMIR and pMIR-IL-32MUT. These results demonstrate that the IL-32 3^′^UTR sequence provided a specific and functional binding site for hcmv-miR-UL112-1.

The regulatory effect of hcmv-miR-UL112-1 on IL-32 protein expression was then examined in HEK293 cells by western blot. IL-32 protein level was significantly reduced in cells transfected with hcmv-miR-UL112-1 in comparison to cells transfected with pSilencer negative control (Figure 3C and D). IL-32 densitometer values were normalized to that of β-actin values. The level of reduction of IL-32 protein was approximately 67% as determined by densitometry (Figure 3D). Our observations confirmed that the expression of endogenous IL-32 protein could be specifically inhibited by ectopically expressed hcmv-miR-UL112-1.

## Discussion

IL-32 is a proinflammatory cytokine and plays a critical role in inflammatory responses. It induces the expression of IL-1β, IL-6, IL-8 and TNF through the p38MAPK, NF-kappa B and AP-1 signaling pathways. Recent reports have highlighted that IL-32 is regulated during viral infection in humans. It has been reported that the IL-32 expression could be induced during infections of influenza A virus, HBV, HCV, HPV and HIV [13-21].

IL-32 levels, detected by ELISA, were 4.1778±1.1663 ng/ml in sera from actively HCMV-infected patients. IL-32 levels in sera from the previously infected group and healthy group were 2.4653±0.8287 ng/ml and 2.4480±0.9162 ng/ml, respectively. IL-32 levels in sera from actively HCMV-infected patients were significantly higher than those in control groups (*F*=23.957, *P*<0.001). However, no significant differences in IL-32 levels were observed between the HCMV previously infected group and healthy group (*P*=0.963). These results demonstrate that IL-32 may be a newly described reactive protein corresponding to active HCMV infection.

HCMV infection can induce the expression of IL-32 at both the mRNA and protein levels in HCMV-infected MRC-5 cells. The peak value of IL-32 mRNA was detected as early as IE stage. The IL-32 protein expression level reached peak value at 6 hpi. The IL-32 protein concentration in HCMV-infected cells at 6 hpi was 10 fold higher than that in uninfected cells. This result demonstrated that the production of IL-32 can be specifically induced by active HCMV infection, while the mechanism that induced the expression of IL-32 during HCMV infection is still unknown. Is the induction of IL-32 mediated only by viral entry or by the expression of IE proteins? Additional experiments would be necessary to provide these answers, and might include the use of UV-irradiated virus. Accumulation of either IL-32 mRNA or protein was not observed following prolonged HCMV-infection process. IL-32 mRNA levels in HCMV-infected cells decreased rapidly in E and L stage to a similar level to that of uninfected cells. IL-32 protein levels decreased in subsequent hours post infection and could only be detected in a small quantity by western blot at 96 hpi. It is hypothesized that certain pathways might be activated to down-regulate or block the expression of IL-32 during HCMV infection.

MiRNAs are the most studied non-coding RNAs in recent years. They regulate gene expression at the post-transcriptional level and act as key regulators in diverse regulatory pathways, including early development, cell differentiation, cell proliferation, metabolism and apoptosis [37-40]. We carried out additional experiments to validate IL-32 mRNA as a target of hcmv-miR-UL112-1. In the presence of pS-miR-UL112-1, 39.27% luciferase activity of pMIR-IL-32UTR was observed to be significantly decreased, and a 67% reduction of endogenous IL-32 protein expression level was measured in HEK293 cells. It was confirmed that hcmv-miR-UL112-1 could specifically repress the expression of IL-32 through the predicted binding site.

To examine the role of hcmv-miR-UL112-1 in regulating IL-32 expression during an active HCMV infection, the kinetics of hcmv-miR-UL112-1 expression in HCMV-infected MRC-5 cells were measured using TaqMan® miRNA assays. The expression of hcmv-miR-UL112-1 could be detected at 24 hpi and then increased gradually with prolonged HCMV infection. This result suggests that hcmv-miR-UL112-1 might function and lead to the decrease of IL-32 levels in E stage during active HCMV infection. The decrease of IL-32 mRNA, but not IL-32 protein level, together with the dramatic increase of hcmv-miR-UL112-1 during the E stage, suggests the possibility that hcmv-miR-UL112-1 may function primarily in the degradation of mRNA. Further studies are needed to address this point.

Through co-evolution with its host, HCMV has evolved effective immune evasion strategies by encoding many immunomodulatory proteins that modulate the host immune response. It is conceivable that HCMV-encoded miRNAs might also be exploited during immune evasion. Hcmv-miR-UL112-1 is expressed with early kinetics. Some functional targets of hcmv-miR-UL112-1 have been shown experimentally to be involved in the modulation of NK cell activation. Stern-Ginossar et al. have identified MICB as a functional target of hcmv-miR-UL112-1 [30]. MICB is a stress-induced ligand of the NK cell activating receptor, NKG2D, which is critical for killing of virus-infected cells by NK cells. Hcmv-miR-UL112-1-mediated down-regulation of MICB, perturbed MICB binding with NKG2D and reduced killing of HCMV infected cells by NK cells. In addition, HCMV IE1, which would increase the TNF-α level by activation of the TNF-α promoter, was then identified as a target of hcmv-miR-UL112-1 by Murphy et al. in 2008 [29]. It has been confirmed that IL-32 induces significant amounts of TNF-α in a dose-dependent manner, and that silencing endogenous IL-32 by short hairpin RNA impairs the induction of TNF-α [2]. In this study, we validated that expression of IL-32 was activated by active HCMV infection and functionally down-regulated by ectopically expressed hcmv-miR-UL112-1 in HEK293 cells. It may be deduced from these results that, besides IE1 and MICB, hcmv-miR-UL112-1 might also synergistically achieve the modulation of NK cell activation through the TNF-α pathway by the down-regulation of IL-32, and immune evasion by HCMV.

## Conclusions

In summary, IL-32 expression in active HCMV infection was firstly investigated in our study. Detailed kinetics of the transcription of hcmv-miR-UL112-1, IL-32 mRNA and its protein expression were determined in HCMV infected MRC-5 cells. Furthermore, IL-32 expression was demonstrated to be functionally down-regulated by ectopically expressed hcmv-miR-UL112-1 in HEK293 cells. Follow up studies will investigate the mechanism of immune evasion by HCMV mediated by hcmv-miR-UL112-1, which has been confirmed to modulate NK cell activation during HCMV infection through post-transcriptional regulation.

## Materials and methods

### Serum samples

Serum samples were obtained from 40 patients with HCMV infection (22 male, 18 female, aged 2.2 ± 0.42 yr) from Shengjing Hospital of China Medical University. All patients were HCMV-IgM positive and HCMV-IgG negative. The viral loads in urine samples from these patients were between 1.793×10^3^ and 4.115×10^7^ HCMV DNA copies per milliliter, as detected by routine QF-PCR (DaAn Gene). Blood samples collected from 32 HCMV-IgM negative individuals (17 male, 15 female, aged 2.7±0.35 yr) at the Medical Examination Centre of Shengjing Hospital were used as controls. According to the HCMV IgG detection results, HCMV IgM negative controls were divided into two groups: Previously HCMV infected group (IgG positive, n=17) and healthy group (IgG negative, n=15). All individuals involved in our study were seronegative for influenza A virus, HBV, HCV and HIV. This study was specifically approved by the Ethical Committee of Shengjing hospital.

### Cell lines

HEK293 cell line was a gift of Dr. Fangjie Chen from Department of Medical Genetics, China Medical University. HEK293 cells were cultured in Dulbecco’s Modified Eagle’s Medium (DMEM) supplemented with 10% fetal bovine serum (FBS), 100 μg/ml penicillin, 100 μg/ml streptomycin sulfate and 2 mM L-glutamine. Human embryonic lung fibroblasts cells, MRC-5, were acquired from Shanghai Institute for Biological Sciences, Chinese Academy of Sciences (CAS). MRC-5 cells were maintained in Modified Eagle’s Medium (MEM) supplemented with 10% FBS, 100 μg/ml penicillin and 100 μg/ml streptomycin sulfate. All cell cultures were maintained at 37°C in a 5% CO_2_ incubator.

### Virus

HCMV clinical strain H was isolated from a urine sample of a 5-month-old infant hospitalized in Shengjing Hospital of China Medical University. Stock virus was propagated in MRC-5 maintained in MEM supplemented with 2% FBS, 100 μg/ml penicillin and 100 μg/ml streptomycin. The supernatant was then harvested, and the aliquots were stored at −80°C before use.

### Plasmid constructs

Luciferase reporter construct pMIR was acquired from Ambion Company. IL-32 3^′^UTR sequence was obtained by RT-PCR from RNA harvested from HCMV infected cells, and inserted into *SpeI* and *HindIII* site of the multiple cloning regions (pMIR-IL-32UTR). A point mutation at corresponding seed region binding site was maintained by using Site-directed Gene Mutagenesis Kit (Beyotime), resulting in GUC to GAA. Sequence predicted to express hcmv-miR-UL112-1 was amplified by PCR directly from HCMV genome. The PCR product was inserted into *BamHI* and *HindIII* sites of pSilencer4.1 (Ambion) to generate a miRNA expression vector pS-miR-UL112-1. All primer sequences used in plasmid construction are listed in Table 1. All constructs were confirmed by DNA sequencing.

**Table 1:** Primers used in plasmid construction and semi-quantitative RT-PCR

**Products**	**Primer sequences**
miR-UL112-1	F: 5^′^-CGCGGATCCTCAGGTACTCGCAGGTGTGC-3^′^
	R: 5^′^-CCCAAGCTTGTTGCCTGGACGCCTGGGCGCGA-3^′^
IL-32UTR	F: 5^′^-GGACTAGTAGATACTGACACCACCTTTGCCCT-3^′^
	R: 5^′^-CCCAAGCTTCATGGTATCTCCCCTGCCAG-3^′^
IL-32MUT	F: ACCTTTGCCCTCCCCGaaACCGCGCACCCACCCTGA
	R: TCAGGGTGGGTGCGCGGTttCGGGGAGGGCAAAGGT
IL-32	F: 5^′^-CATGAATTCCATGCTTCCCGAAGG-3^′^
	R:5^′^-CTACTCGAGGTATCTTCATTTTGAGGATTG-3^′^
β-actin	F: 5^′^-CTCCATCCTGGCCTCGCTGT-3^′^
	R: 5^′^-GCTGTCACCTTCACCGTTCC-3^′^

Note: sequences recognized by restriction en donuclases are down lined.

### Enzyme-linked immunosorbent assay

The concentration of IL-32 in serum samples were detected using human IL-32 ELISA Kit (Cusabio) according to the manufacture’s protocols. The absorbance value at wavelength 450 nm was measured. The IL-32 concentrations were calculated from the standard curve.

### Semi-quantitative RT-PCR analysis

MRC-5 cells were inoculated with H strain at 3–5 multiplicity of infection (m.o.i.). Infections were carried out under IE, E and L conditions respectively. For preparation of IE RNA, CHX (Sigma) (100 μl/ml) was added to the culture medium 1 hour before infection and the cells were harvested at 24 hpi. For E RNA, DNA synthesis inhibitor phosphonoacetic acid (PAA) (Sigma) (100 μl/ml) was added to the medium immediately after infection, and the cells were harvested at 48 hpi. L RNA and uninfected cellular RNA was derived from infected (at 96 hpi) and uninfected cells, respectively. Total RNA was isolated using Trizol reagent (TaKaRa), treated with DNase Iand then reverse-transcribed with MLV reverse transcriptase and random primers (TaKaRa). PCR was performed for 24 cycles in 50 μl reactions with the IL-32 specific primer pairs listed in Table 1. mRNA of β-actin was amplified for normalization in all reactions. The primers for IL-32 amplification were designed for detecting all known isoforms of human IL-32 based on the sequences from Gnebank database (NM_001012631.1-001012636.1, NM_004221.4 and NM_001012718.1). The PCR products were analyzed by electrophoresis on 2% agarose gel containing ethidium bromide.

### miRNA extraction and TaqMan assays

Total RNA was extracted from HCMV infected cells at different time points using the mivVana miRNA isolation kit (Ambion). We measured hcmv-miR-UL112-1 in all samples using TaqMan® miRNA assays (MIMAT0001577 for hcmv-miR-UL112-1, ABI). U6 expression was used to normalize the expression of hcmv-miR-UL112-1. HCMV uninfected MRC-5 cells were used as negative control. Fold difference for hcmv-miR-UL112-1 expression was calculated using the equation 2^ΔΔct^, where ΔCt = Ct (hcmv-miR-UL112-1) – Ct (U6 control) and ΔΔCt = ΔCt (HCMV infected MRC-5) – ΔCt (MRC-5). The measurements were done in triplicates and the results are presented at the means ± S.D.

### Luciferase assay

MRC-5 cells were plated in 24-well plate at a density of 4.0×10^5^ cells per well and grown to reach about 80% confluence at the time for transfection. 200 ng pMIR-IL-32UTR/pMIR-IL-32MUT plasmid or an empty vector control was co-transfected with 400 ng pS-miR-UL112-1 or pS-neg control into the cells by using lipofectamine 2000 reagent (Invitrogen) according to the manufacture’s protocol. 200 ng renilla luciferase plasmid (Promega) was introduced into each co-transfected cells as an internal control plasmid at the same time. Cells were collected 48 hours post transfection and luciferase activity levels were measured using the Dual luciferase reporter assay system (Promega) according to the manufacture’s guidelines. All measurements were done in triplicates and signals were normalized for transfection efficiency to the internal renilla control. The results are presented at the means ±S.D.

### Western blot analysis

Western blot analyses were performed to detect the IL-32 levels in HCMV infected MRC-5 cells and hcmv-miR-UL112-1 over expressed HEK293 cells respectively. MRC-5 cells were inoculated with H strain at 3–5 m.o.i. Cells were harvested at different time points post infection (6 h, 24 h, 48 h, 72 h and 96 h). HEK293 cells were transfected with 8 μg pS-miR-UL112-1 or pS-neg control using lipofectamine 2000 (Invitrogen) and were incubated at 37°C for 48 hours.

Proteins of the cells described above were prepared by suspending cells in lysis buffer, followed by centrifugation. Concentrations of proteins in supernatant were quantified using protein assay kit (Beyotime). Protein was separated by 10% acrylamide gel electrophoresis and transferred onto a nitrocellulose membrane. Western blot analysis was performed using IL-32 antibody (abcam). Immunoblots were visualized with ECL detection system. Sample loading was normalized by quantities of β-actin detected parallel.

### Statistical analysis

All experiments were reproducible. The results are presented at the means ± S.D. Student’s *t* test and *F* test was used to determine statistical significance. Differences were considered statistically significant at value of *P*≤0.05.

## Competing interests

The authors declare that they have no competing interests.

## Authors’ contributions

YJH carried out the semi-quantitative RT-PCR analysis, western blot analysis and Statistical analysis. YQ and RH carried out the plasmids construction and luciferase assay. QR as the corresponding author designed the study and corrected the manuscript. YPM and YHJ carried out the preparation of virus and cell lines. ZRS carried out the serum samples collection and ELISA detection. All authors have read and approved the final manuscript.
